# Diarylureas: New Promising Small Molecules against *Streptococcus mutans* for the Treatment of Dental Caries

**DOI:** 10.3390/antibiotics12010112

**Published:** 2023-01-07

**Authors:** Domenico Iacopetta, Jessica Ceramella, Alessia Catalano, Assunta D’Amato, Graziantonio Lauria, Carmela Saturnino, Inmaculada Andreu, Pasquale Longo, Maria Stefania Sinicropi

**Affiliations:** 1 Department of Pharmacy, Health and Nutritional Sciences, University of Calabria, 87036 Arcavacata di Rende, Italy; 2 Department of Pharmacy-Drug Sciences, University of Bari “Aldo Moro”, 70126 Bari, Italy; 3 Department of Chemistry and Biology, University of Salerno, Via Giovanni Paolo II, 132, 84084 Fisciano, Italy; 4 Department of Science, University of Basilicata, 85100 Potenza, Italy; 5 Departamento de Química, Universitat Politècnica de València, Camino de Vera s/n, 46022 Valencia, Spain; 6 Unidad Mixta de Investigación UPV-IIS La Fe, Hospital Universitari i Politècnic La Fe, Avenida de Fernando Abril Martorell 106, 46026 Valencia, Spain

**Keywords:** antimicrobials, biofilms, bis-aryl ureas, dental caries, diarylureas, diphenylureas, quorum sensing, *Streptococcus mutans*, *Streptococcus sobrinus*

## Abstract

Dental caries is a biofilm-mediated disease that represents a worldwide oral health issue. *Streptococcus mutans* has been ascertained as the main cariogenic pathogen responsible for human dental caries, with a high ability to form biofilms, regulated by the quorum sensing. Diarylureas represent a class of organic compounds that show numerous biological activities, including the antimicrobial one. Two small molecules belonging to this class, specifically to diphenylureas, BPU (1,3-bis[3,5-bis(trifluoromethyl)phenyl]urea) and DMTU (1,3-di-*m*-tolyl-urea), showed interesting results in studies regarding the antimicrobial activity against the cariogenic bacterium *S. mutans*. Since there are not many antimicrobials used for the prevention and treatment of caries, further studies on these two interesting compounds and other diarylureas against *S. mutans* may be useful to design new effective agents for the treatment of caries with generally low cytotoxicity.

## 1. Introduction

Dental caries, also known as tooth decay, is one of the most frequent chronic diseases of people in the world [1]. People are susceptible to this disease throughout their whole life and its early clinical signs start with a small patch in the surface of the tooth enamel or, sometimes, occur between the teeth fissures. Then, caries spreads in the bone body and reach the bulb of the tooth, the enamel weakens and collapses to produce a cavity, developing to a destruction status. The occurrence of dental caries is considerable [2]. Indeed, the results of the Global Burden of Disease Study 2017 showed that, among 328 diseases, permanent dental caries ranked first [3,4]. Early childhood caries (ECC) is one of the most common infectious diseases linked to biofilm formation in children around the world [5]. Dental caries develops through a complex interaction between acidic by-products from bacteria and fermentable carbohydrate, and a myriad of other factors such as teeth and saliva [6]. The pathogenic bacteria generally related to dental caries are the Gram-positive mutans streptococci (*Streptococcus mutans*, *S. sobrinus*, *S. rattus*, *S. cricetus*, and *S. downei*) and lactobacilli species present in the supragingival plaque [7]. The main bacterial strains involved in dental caries are considered *S. mutans* and *Lactobacillus acidophilus*: the former is likely responsible of the decay process as a causative factor and the latter as a progressive one [8]. The presence of *Candida* spp., specifically *C. albicans*, in the oral environment is an additional factor that needs to be considered when evaluating risks to caries [9,10]. Moreover, the association of *C. albicans* with *S. mutans* and *Actinomyces viscosus* has been shown to significantly increase the cariogenic virulence of biofilm in dental caries [11,12]. Dental caries is principally combated with the daily oral hygiene by diverse strategies, which even today are not yet totally effective [13]. Fluoride-containing toothpastes, used to prevent the insurgence of dental caries, led to reductions in caries experience in many countries [14]. Indeed, in the presence of fluoride, calcium fluoride (CaF_2_) is formed on the enamel surface and serves mainly as a physical barrier able to prevent interaction between acids and the enamel [15]. However, several reports related to the excess fluoride toxicity are described in the literature [16]. In addition to fluoride, the use of antimicrobials to treat dental caries is often needed [17]. Moreover, this practice may lead to undesirable effects, including pigmentation and bacterial resistance [18], which led to a shortage of options when choosing effective treatment agents. The broad-spectrum antimicrobial agents, such as chlorhexidine, are widely used to control cariogenic pathogens. However, the role of chlorhexidine in preventing dental caries is controversial [19,20]. Moreover, it has several limits, including taste confusions, mucosal soreness, tooth discoloration, disruption of the oral microbial equilibrium, and drug resistance. In this context, some interesting antimicrobial small molecules have been described as promising tools for the control of *S. mutans*, due to good antimicrobial activity, high selectivity, and low toxicity [21,22,23]. BPU and DMTU (Table 1) are two small molecules belonging to the diarylureas that have shown interesting activity against dental caries. BPU showed antimicrobial activity against the cariogenic bacterium *S. mutans* [24], whereas DMTU was able to inhibit the biofilm formation in vivo, targeting the quorum sensing (QS) systems [25]. Recently, photodynamic therapy (PDT), based on a photochemical reaction acting selectively, has shown promising results for the inactivation of microorganisms related to dental caries. This reaction, occurring between a photosensitizer (erythrosine, phloxine B, curcumin and fotoenticine) and an appropriate light, can be applied on the target [26,27]. Finally, the COVID-19 pandemic [28], followed by the post-COVID [29], has totally changed daily habits of people with a serious impact on oral hygiene, eating habits, and oral health in the provision of emergency dental and routine oral health services and oral hygiene maintenance at home [30,31].

## 2. Dental Caries

Dental caries is a chronic infectious disease characterized by localized destruction of dental hard tissues. Several factors are involved in the development of dental caries, such as the attachment of bacteria to the surface of the teeth, the formation of biofilms on the teeth (namely the dental plaque), and the local demineralization of the tooth surface initiated by the acidic by-products from bacterial fermentation of carbohydrates [32]. Dental tissues are made up of enamel, dentin, and cementum, and represent the oral solid surfaces covered by a film to which the microbial cells may attach. The pathogenic microorganisms link one to each other on the surface of teeth and originate a matrix of exopolysaccharide within which cells grow, forming biofilm [32]. The resulting biofilm formed is the dental plaque that exposes teeth and gingival tissues to high concentrations of microbial metabolites resulting in dental disease [33]. The disease may develop in both the crowns and roots of teeth, and it can occur in infancy as an aggressive tooth decay that damages the primary teeth of infants and young children [34]. Although simple and cost-effective preventive measures are easily available, untreated caries of permanent teeth impaired 3.5 billion people worldwide in 2019, making it the most diffuse health condition affecting mankind [35,36]. It is estimated that around 2 million people worldwide suffer from caries in permanent teeth and 520 million children suffer from caries in the firstborn teeth [37]. A systematic review with meta-analysis using the WHO diagnostic criteria was reported, describing studies published from 1960 to 2019, showing that ECC prevalence varied widely depending on country differences rather than continent or change over time [38].

Dental caries is formed by *S. mutans* through the glucosyltransferase (GTF) activity, thus, the molecules targeting GTFs and inhibiting the *S. mutans* biofilm formation, probably, can prevent dental caries [39]. Moreover, *S. mutans* synthesize exopolysaccharide (EPS) using GTFs, resulting in the formation of biofilms on the tooth surface. The EPS matrix has been considered as a virulence determinant of cariogenic biofilm. Therefore, targeting EPS metabolism could be useful in preventing cariogenic biofilm formation, as well [40].

### 2.1. Diagnostics and Risk Factors

The main issues to be addressed in the development of caries are the diagnosis and identification of patients with dental caries [41], risk and treatment difficulty assessment, and treatment planning [42]. The levels of salivary proteins, especially alpha-amylase, acidic proline-rich protein-1 (PRP-1), histatin-5 (Hst-5), lactoperoxidase, mucin-1 (MUC1), carbonic anhydrase VI (CA6), proteinase-3 (PR3), and statherin, have been suggested as biomarkers for caries diagnosis [43]. The most common mutans streptococci are *S. mutans* and *S. sobrinus* [44,45]. *S. sobrinus* is implicated in caries development in cases where it appears to be independent of *S. mutans* [46]. Other mutans streptococci, such as the Gram-positive bacterium *Streptococcus sanguinis*, previously known as *S. sanguis*, are typically associated with healthy plaque biofilm [47], even though the cariogenic potential is lower than *S. mutans* [48]. The presence of plaque rich in bacteria (specifically *S. mutans*) directly eradicates the enamel layer by dissolving tooth minerals (especially hydroxyapatite, Ca_10_(PO_4_)_6_(OH)_2_), resulting in caries. As the lesion progresses to the deeper dentin, anaerobic species begin to thrive and a transition occurs from predominantly facultative Gram-positive bacteria to anaerobic Gram-positive rods and cocci and Gram-negative anaerobic bacteria, including *Fusobacterium nucleatum*, *Porphyromonas gingivalis*, and *Prevotella intermedia*, which are present primarily in the subgingival plaque and known to be periodontal pathogens [49,50]. The persistence of the acidic condition promotes the proliferation and the attachment of other acidogenic and aciduric pathogens, such as *C. albicans*, non-mutans streptococci, *Lactobacillus*, and *Actinomyces* [51,52]. Moreover, Garcia et al. (2021) [53] reported that the expression of collagen-binding proteins (Cbps) confers *S. mutans* the ability to bind more eagerly collagen-rich tooth surfaces, including dentin and roots, invading oral epithelial cell lines, thus contributing to *S. mutans* expansion and caries risk. Particularly, Cbp^+^ *S. mutans* is associated with increased caries incidence and poor outcomes in children with ECC. Cbp^+^ *S. mutans* and *C. albicans* are intimately associated with caries recurrence, contributing to the establishment of recalcitrant biofilms. The analysis of salivary microbiome showed that *Streptococcus parasanguinis* was overrepresented in children with caries recurrence.

### 2.2. Streptococcus mutans

*S. mutans* was identified by Clarke in 1924 in the laboratories of Sir Almroth Wright at St Mary’s Hospital with a grant from the Medical Research Council [54]. Since then, it has been the focus of stringent research efforts due to its involvement in caries initiation and progression. *S. mutans* is now well recognized as the major causative factor of dental caries because of the aciduric and acid production properties, as well as the ability to synthesize glucans and form biofilms [40]. Mutans streptococci are strong acid producers and hence cause an acidic environment increasing the risk for cavities. Indeed, the appearance of *S. mutans* in the tooth cavities is followed by caries after 6–24 months [55]. *S. mutans* is a facultative anaerobic Gram-positive coccus, which encodes GFTs that can convert the dietary starches to extracellular polymers such glucan. The latter may bind to the dental pellicle, then allowing the attachment of other bacteria, including *S. sanguinis*, *S. oralis*, and *Lactobacillus* sp. [56]. GTFs enhance the biofilm formation and promotes colonization of cariogenic bacteria by generating biofilm EPSs, the key virulence property in the cariogenic process. Recently, the existence of new *S. mutans* emerging strains has been suggested by analyzing multi-locus sequence typing from Iranian and Afghan children [57,58].

### 2.3. Prevention of Dental Caries

Since the 1950s, epidemiological studies provided the basis for the use of fluoride in caries prevention bringing the industries to produce effective fluoride-containing toothpastes and other fluoride vehicles. Fluoride varnish effectively prevents ECC, is easily acceptable among children, and enhances the resistance of enamel dissolution by acid by forming fluorapatite, Ca_10_(PO_4_)_6_F_2_ [59,60]. Fluoride concentrated in plaque and saliva may inhibit the demineralization of dental hard tissue and the conversion of carbohydrates to acids by cariogenic bacteria, thus affecting the production of adhesive polysaccharides by bacteria [61]. Caries preventive effects of fluoride are almost exclusively topical and the first one was sodium fluoride (NaF) (1941), followed by stannous fluoride (SnF_2_) (1947), acidulated phosphate fluoride (APF) (1963), varnish containing fluoride (1964) and amine fluoride (1967) [62]. However, the presence of fluoride is currently still highly controversial, as it could have negative health effects. Fiorillo et al. [63] assessed that it did not present important contraindications, except those commonly reported for fluoride, whereas showing significant effects against erosion the biofilm formation, gingival inflammation and favoring recalcification of the enamel. However, toxicity related to excess fluoride has been often reported [64]. The European Academy of Pediatric Dentistry supports the daily use of fluoride for the prevention and control of dental caries in children. For children at least seven years old, parents must be informed to apply an age-related quantity of toothpaste and supervise toothbrushing by children. The use of fluoride must be balanced between the risk of caries and the risks of potential adverse effects [65,66]. Since 1969, silver diamine fluoride [67] has been used for primary teeth caries in children, specifically by preventing cavities and fissure caries of the erupting permanent molars. Moreover, it has been suggested for preventing root caries in elderly people, thus receiving the approval by US Food and Drug Administration (FDA) in 2014 [68]. Silver nanoparticles (AgNPs) and nanosilver fluoride are also under study as caries arresting agents [69,70].

### 2.4. Treatment Strategies

#### 2.4.1. Antimicrobials

Along with mechanical removal, including toothbrushing and using dental floss, the topical application of antimicrobial agents is requested as adjuvant in the control of caries, particularly for high-risk populations. The strategies used against *S. mutans* are biofilm disruption [71] and targeting QS [72,73,74]. Chlorexidine is a cationic polybiguanide, which was one of the first antiseptics suggested for use in dental caries demonstrating high effectiveness [75]. Up to now, chlorhexidine remains the “gold standard” of antiplaque agents, being active against Gram-positive and Gram-negative bacteria, facultative anaerobes, aerobes, and fungi by disrupting the inner cytoplasmic membrane [76]. Its mechanism of action is related to the block of the acidic groups of glycoproteins present in saliva, thus reducing plaque adhesion [77]. However, it has been reported that it may cause genotoxicity by damaging DNA in leukocytes, kidney cells, and oral mucosal cells and inducing cellular apoptosis [78,79]. The potential use of Cannabis as antibacterial has recently been described. One of the minor phytocannabinoid, represented by the non-psychoactive cannabigerol, has shown anti-bacterial activity against *S. mutans*, also preventing the lowering in pH caused by *S. mutans* [80]. Moreover, several antimicrobial peptides showed bactericidal activity against *S. mutans* [81] and some of them prevented bacterial adhesion [82].

#### 2.4.2. Diarylureas

Among the antimicrobials studied, an interesting activity was found for diarylureas that possess a privileged structure endowed with numerous activities [83]. The well-known activity is the anticancer one [84,85], with some compounds, such as sorafenib, regorafenib, and tivozanib, being used in therapy for various cancers, beyond other interesting recent studies at this regard [86,87]. Additionally, and not less important, these compounds have demonstrated numerous other pharmacological activities, such as antiviral, anti-inflammatory, antiplatelet, antiparasitic, and specifically antimicrobial actions, also against MRSA [88,89,90]. Recently, the anti-pseudo-allergic properties of a series of diarylureas, specifically diphenyl ureas, have been demonstrated [91]. Particularly, the small molecules BPU and DMTU have been studied against *S. mutans*, giving interesting results.

BPU (Table 1, CAS number: 3824-74-6, Schreiner’s Catalyst) is a commercial small molecule, belonging to diarylureas, firstly studied as antimicrobial in 2015 by Nelson et al. [92], also used in organic chemistry as catalyzer [93,94,95,96]. It must not be confused with benzoylphenylureas, since it possesses the same acronym [97,98]. In the study by Nelson, a series of diarylureas, isolated by using a high-throughput screen (HTS), bearing fluorine atoms was tested against *Escherichia coli* and *S. mutans*. It was demonstrated that, when used in combination with fluoride, they enhanced fluoride toxicity in *Escherichia coli* and *S. mutans*. Interestingly, among this series, compound **19** (successively named BPU) exhibited the greatest ability to increase fluoride toxicity on *S. mutans*. The higher antimicrobial ability was related to the trifluoromethyl substituents on the aryl rings, which facilitate fluoride uptake and/or retention [92]. Additional studies are needed to demonstrate the exact mechanism of action of this compound. However, it could disrupt the bacterial membrane, thus allowing fluoride to more rapidly enter cells, or inhibit its transport by interfering with membrane potential. Moreover, BPU was previously studied as a compound able to promote the transport of chloride out of lipid vesicles [99]. Recently, Liao et al. [24] demonstrated that BPU was capable of inhibiting planktonic growth, as well as biofilm formation of *S. mutans* UA159 in vitro. However, the exact mechanism remains unclear. A molecular modeling study predicted that it may strongly bind vital enzymes (3AIC and 2ZID) involved in EPS synthesis. Moreover, the transcriptomic study has revealed alterations in stress response and nitrogen metabolism in *S. mutans* treated with BPU. In particular, the expression of genes encoding 3AIC and 2ZID (*gtfC* and *dexB*) and other genes with associated functions (*gtfB*, *ftf* and *dexA*) were upregulated in *S. mutans* treated with BPU [24]. Furthermore, drug-loaded poly(lactic-co-glycolic acid) (PLGA) nanoparticles containing BPU have shown interesting results in studies on planktonic *S. mutans* as well as *S. mutans* biofilms. A biphasic drug release in vitro was observed, which was higher in an acidic medium (pH 4.5) than in a neutral one (pH 7.4). The drug-loaded PLGA nanoparticles markedly inhibited the growth and lactic acid production of planktonic *S. mutans* and of *S. mutans* biofilms, while showing negligible cytotoxic activity against the human oral squamous cell carcinoma (OSCC) cell line CAL27 [100]. This compound had been also studied as activator of the eIF2a kinase heme regulated inhibitor, suggesting its anticancer potential. It showed marked inhibition of ternary complex (TC) formation between eukaryotic translation initiation factor 2 (eIF2), GTP, and initiator methionine tRNA (tRNAMet i) [101].

DMTU (Table 1) was first studied by Kaur et al. (2016) [102], who synthesized the compound ComAI (successively named DMTU), as inhibitor of ComA, a bacteriocin associated ABC transporter, involved in the QS of *S. mutans*. The synthesis, biological, and molecular modeling studies were carried out on DMTU. Then, some analogues (ComAI^1^, ComAI^2^, ComAI^3^, and ComAI^4^) were studied, showing good biofilm inhibition, and exhibiting a potent synergy with low concentrations of fluoride (31.25–62.5 ppm) in the inhibition of the biofilm formation, even though showing no antibacterial activity. Biological assays were carried out on *S. mutans* MTCC 497, a wild type strain (WT) NG8 and two clinical isolates of *S. mutans* 4SM (multidrug resistant) and 5SM (fluoride resistant). ComAI and ComAI^1^ were the most active in biofilm inhibition, especially against strains 4SM and MTCC497 (IC_50_ = 0.39 nM and 3.75 μM for ComAI and IC_50_ = 0.89 and 1.89 μM for ComAI^1^, respectively). Contrarily, no biofilm inhibition or eradication were induced in 5SM strain by any of the synthesized compounds. Considering the better biofilm inhibiting activity of ComAI and ComAI^1^, they were titled as best amongst all the synthesized compounds. In a successive paper by Kaur et al. (2017) [103], a more in-depth study was carried out on ComAI and ComAI^1^ by evaluating their activity in vivo, alone or in combination with fluoride. The compound ComAI inhibited the development of the *S. mutans* biofilm in a Wistar rat model for dental caries. Histopathological analyses evidenced the appearance of lesions on dentine in infected subjects while that of the treated rodents remained intact and healthy. Moreover, a decrease in inflammatory markers (TNF-α, CRP, IL-1 and IL-6) in rodents’ blood and liver samples was ascertained after treatment with ComAI. The treatment of MTCC 497 and SM4 strains with DMTU determined the downregulation of the genes implicated in competence development and bacteriocin production. The authors speculated that ComAI, also defined DMTU in this paper, is an efficient antibiofilm and anti-inflammatory agent that can enhance the cariostatic properties of fluoride. Moreover, ComAI and ComAI^1^ do not have any quantitative cytotoxic effect against liver hepatocellular carcinoma (Hep G2) cell lines, as revealed by MTT assay. Recently, Kalimuthu et al. (2020) [25] have underlined the importance of DMTU by developing a multispecies oral biofilm model, consisting of an early colonizer *Streptococcus gordonii*, a bridge colonizer *Fusobacterium nucleatum*, and late colonizers *Porphyromonas gingivalis* and *Aggregatibacter actinomycetemcomitans*. DMTU was shown to inhibit multispecies biofilms, by disrupting them while showing no bactericidal effect. Mechanistic studies revealed the downregulation of biofilm and a number of virulence genes of the periodontal pathogen *P. gingivalis* in polymicrobial communities. The study evidenced the potential of DMTU to inhibit polymicrobial biofilm communities and their virulence in a dose-dependent manner. Thus, DMTU was suggested as a promising prophylactic as well as therapeutic agent.

Several new studies are related to analogues of triclocarban, an antimicrobial ingredient belonging to diarylureas, found in consumer and industrial products. However, its use has been banned in over-the-counter hand and body washes by the U.S. Food and Drug Administration because of its toxicity and endocrine disruptive properties [104]. Thus, new studies are addressed to its analogues as antimicrobials, some of them against *S. mutans*. Pujol et al. (2018) [105] reported a series of pentafluorosulfanyl-containing triclocarban analogs with potent in vitro antimicrobial activity towards *S. mutans*. Among them, compounds **5**, **6**, **9**, **10**, **11**, and **14** showed high activity against wild-type *S. mutans* CECT 479 (ATCC 25175), with minimum inhibitory concentration 50% (MIC_50_) values of 0.5 µg/mL. Compounds **10**, **11**, and **14** were the less cytotoxic against J-774A.1 murine macrophages cells compared to the parent compound triclocarban (CC_50,_ cytotoxic concentration 50% = 20.6, 33.5; 33.8 µg/mL versus 14.5 µg/mL of triclocarban). Other diarylureas analogs of triclocarban have been recently reported by our research group as antimicrobial agents against Gram positive bacteria [106,107]. It would be interesting to study these compounds against *S. mutans*.

**Table 1:** Structures of diarylureas showing activity against *S. mutans*.

Structure	Name or Number	Ref	Mechanism of Action
** 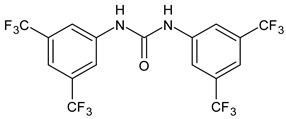 **	Schreiner’s Catalyst	Sigma Aldrich (Italy)	
	**19**	Nelson et al., 2015 [92]	- Increase in fluoride toxicity on *S. mutans*, facilitating the transport of fluoride into bacteria.
	BPU	Liao et al., 2022 [24]	- Inhibition of planktonic growth and biofilm formation of *S. mutans*. - Disruption of the ability of biofilms to synthesize EPS and upregulation of the expression of genes encoding 3AIC and 2ZID (gtfC and dexB) and other genes (gtfB, ftf, and dexA) with associated functions in *S. mutans*.
** 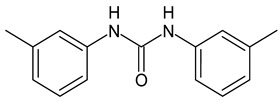 **	ComAI	Kaur et al., 2016 [102]	- Inhibition of ComA (in silico studies). - Inhibition of biofilms in different strains (MTCC 497, WT, 4SM) with IC50 ranging from 0.39 to 14.9 µM. Synergistic and additive effects in combination with fluoride.
	DMTU	Kaur et al., 2017 [103]; Kalimuthu et al., 2020 [25]	- Inhibition in vivo of the biofilm development of *S. mutans* in a Wistar rat model for dental caries. - Downregulation of genes located downstream to comA in MTCC 497 (except for immA and immB genes that were upregulated). - Decrease in inflammatory markers expression (TNF-α, CRP, IL-1 and IL-6) in liver and plasma samples.
** 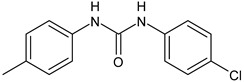 **	ComAI^1^	Kaur et al., 2016 [102]	- Inhibition of ComA (in silico studies). - Biofilm inhibition in different strains (MTCC497, WT, 4SM) with IC50 ranging from 0.41 to 14.9 µM. Synergistic and additive effects in combination with fluoride.
** 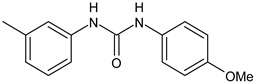 **	ComAI^2^	Kaur et al., 2016 [102]	
** 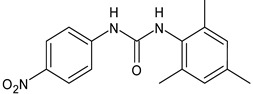 **	ComAI^3^	Kaur et al., 2016 [102]	
** 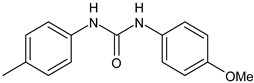 **	ComAI^4^	Kaur et al., 2016 [102]	
** 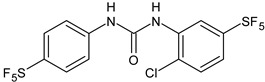 **	**5**	Pujol et al., 2018 [105]	- High activity against wild-type *S. mutans* CECT 479 (ATCC 25175) with MIC50 = 0.5 µg/mL. - Low cytotoxicity against J-774A.1 murine macrophages cells compared to the parent compound triclocarban.
** 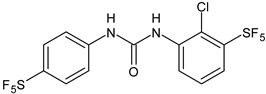 **	**6**	Pujol et al., 2018 [105]	
** 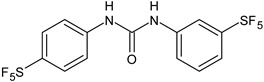 **	**9**	Pujol et al., 2018 [105]	
** 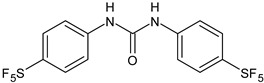 **	**10**	Pujol et al., 2018 [105]	
** 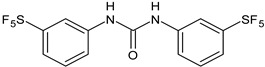 **	**11**	Pujol et al., 2018 [105]	
** 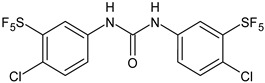 **	**14**	Pujol et al., 2018 [105]	

## 3. Conclusions

Dental caries and dental plaque are common diseases in the world caused by a mixture of microorganisms and food debris. Caries and ECC are major issues particularly in part of society characterized by a low socioeconomic status and a limited access to healthcare. Moreover, even though young individuals can be affected by root caries, the prevalence is higher with increasing age. Indeed, in developed countries, the growing elderly population shows an increasing incidence and significance of root caries. Several approaches are used to prevent and treat this disease, as a strong promotion and attention to the oral hygiene and an appropriate diet directed to eliminate or diminish the frequent consumption of carbohydrate-rich foods or beverages with a high content of sugar. Regarding the daily oral hygiene, several toothpastes containing fluoride and antimicrobials such as chlorhexidine have been proposed. Nevertheless, given the toxicity or the not fully confirmed antimicrobial activity of these compounds, new agents acting against *S. mutans* are needed. BPU and DMTU are small molecules belonging to the class of diarylureas, particularly diphenylureas, which have demonstrated interesting activity against the cariogenic bacterium *S. mutans*, the most important bacteria related to the formation of dental caries. The former acts via the inhibition of biofilm formation, while the latter acts on QS. It is interesting to note that these two compounds are symmetrical diarylureas. Thus, it could be interesting to study other analogs of these compounds bearing a symmetrical structure against *S. mutans*.

## Data Availability

Not applicable.
